# Activation of CO_2_ assimilation during photosynthetic induction is slower in C_4_ than in C_3_ photosynthesis in three phylogenetically controlled experiments

**DOI:** 10.3389/fpls.2022.1091115

**Published:** 2023-01-04

**Authors:** Lucía Arce Cubas, Richard L. Vath, Emmanuel L. Bernardo, Cristina Rodrigues Gabriel Sales, Angela C. Burnett, Johannes Kromdijk

**Affiliations:** ^1^ The University of Cambridge, Department of Plant Sciences, Cambridge, United Kingdom; ^2^ University of the Philippines Los Baños, Institute of Crop Science, College of Agriculture and Food Science, College, Laguna, Philippines

**Keywords:** C_4_ photosynthesis, C_3_ photosynthesis, photosynthetic induction, CO_2_ assimilation, photorespiration, non-steady state, light response

## Abstract

**Introduction:**

Despite their importance for the global carbon cycle and crop production, species with C_4_ photosynthesis are still somewhat understudied relative to C_3_ species. Although the benefits of the C_4_ carbon concentrating mechanism are readily observable under optimal steady state conditions, it is less clear how the presence of C_4_ affects activation of CO_2_ assimilation during photosynthetic induction.

**Methods:**

In this study we aimed to characterise differences between C_4_ and C_3_ photosynthetic induction responses by analysing steady state photosynthesis and photosynthetic induction in three phylogenetically linked pairs of C_3_ and C_4_ species from *Alloteropsis*, *Flaveria*, and *Cleome* genera. Experiments were conducted both at 21% and 2% O_2_ to evaluate the role of photorespiration during photosynthetic induction.

**Results:**

Our results confirm C_4_ species have slower activation of CO_2_ assimilation during photosynthetic induction than C_3_ species, but the apparent mechanism behind these differences varied between genera. Incomplete suppression of photorespiration was found to impact photosynthetic induction significantly in C_4_ *Flaveria bidentis*, whereas in the *Cleome* and *Alloteropsis* C_4_ species, delayed activation of the C_3_ cycle appeared to limit induction and a potentially supporting role for photorespiration was also identified.

**Discussion:**

The sheer variation in photosynthetic induction responses observed in our limited sample of species highlights the importance of controlling for evolutionary distance when comparing C_3_ and C_4_ photosynthetic pathways.

## Introduction

Photosynthesis is the foundation of life on earth, the source of food, oxygen, and most of our energy. A particularly successful adaptation to the ancestral form is C_4_ photosynthesis, which despite its complexity has independently arisen in at least 66 lineages of angiosperms and appeared in 19 unrelated plant families (Sage, 2004; Kellogg, 2013). Although only 3% of flowering species use the C_4_ pathway, C_4_ species represent 23% of global carbon fixation (Still et al., 2003), and maize and sugar cane are two of the four crops that account for half of the world’s crop production (FAO, 2020). Despite the undeniable importance of C_4_ species, there is comparatively less focus on improving C_4_ performance – photosynthetic induction has consistently been flagged as a source of inefficiency in C_4_ species relative to C_3_ ones (Sage and McKown, 2006; Slattery et al., 2018; Sales et al., 2021), yet a lack of knowledge on the specifics of the C_4_ induction response persists due to limited understanding of variation in photosynthetic induction between C_3_ and C_4_ photosynthesis, as well as across different C_4_ species.

Most C_4_ species display ‘Kranz’ anatomy, in which mesophyll (M) and bundle sheath (BS) cells are arranged concentrically around the leaf veins. Unlike in C_3_ species, where initial CO_2_ fixation and assimilation processes occur within the same cell, in C_4_ species these activities are typically partitioned between M and BS cells. In the cytosol of M cells, equilibrium between CO_2_ and bicarbonate is rapidly established by carbonic anhydrase. Bicarbonate is then fixed by phosphoenolpyruvate carboxylase (PEPC) into 4-carbon molecules that are further reduced before diffusing into the BS, where they are decarboxylated to release CO_2_ around ribulose 1,5-biphosphate carboxylase/oxygenase (Rubisco), the central enzyme in carbon fixation (Leegood, 2002). The C_4_ carbon concentrating mechanism (CCM) thus enhances photosynthesis and suppresses Rubisco’s oxygenase activity and resulting photorespiration – the phospho-glycolate salvaging pathway that consumes energy and reducing equivalents, and releases CO_2_. However, the operation of the CCM has an energetic cost, and C_4_ species require additional ATP for PEP regeneration on top of the energetic demands of the C_3_ cycle (Yin and Struik, 2018).

The increased efficiency of the C_4_ pathway relative to the C_3_ ancestral state is especially apparent under constant high light (Wang et al., 2012). However, during changes in light intensity, some C_4_ species display impaired carbon assimilation in comparison to C_3_ species (Kubásek et al., 2013; Slattery et al., 2018; Li et al., 2021). Decreases in photosynthetic efficiency during light induction (in response to an increase in light intensity) occur irrespective of photosynthetic pathway and are often explained by lags in regeneration of ribulose-1,5-biphosphate (RuBP) within the C_3_ cycle, Rubisco activation, and stomatal opening (Pearcy and Seemann, 1990; Pearcy, 1990; Sassenrath-Cole and Pearcy, 1992; Mott and Woodrow, 2000). In addition, C_4_ photosynthesis requires synchronous operation of C_3_ and C_4_ cycles and any loss of coordination during induction could lead to reduced efficiency of carbon fixation in C_4_ species. Faster activation of the CCM relative to C_3_ cycle activation in the BS may result in diffusional leakage of highly concentrated CO_2_ out of the permeable BS back into M cells and a raised energetic cost for CO_2_ assimilation, as recently suggested by Wang et al. (2022) and Lee et al. (2022). A 30-60% increase in BS leakiness of CO_2_ during photosynthetic induction relative to steady state photosynthesis has been identified in maize and sorghum (Wang et al., 2022). This suggests that the C_3_ cycle in some C_4_ species is slower to activate than the C_4_ CCM. Alternatively, the C_4_ cycle could be the limiting factor during induction due to the need to build up metabolite gradients for the shuttling of CCM intermediates between M and BS cells. If so, the reduced supply of CO_2_ to the BS would lead to weaker suppression of photorespiration, and a temporary disconnect between photosynthetic electron transport and CO_2_ fixation (Sage and McKown, 2006; Kromdijk et al., 2010; Slattery et al., 2018). Although incomplete suppression of photorespiration can reduce photosynthetic efficiency, photorespiratory metabolite pools have also been suggested to help prime the C_4_ cycle (Kromdijk et al., 2014; Stitt and Zhu, 2014; Schlüter and Weber, 2020; Medeiros et al., 2022). Higher relative photorespiratory rates appear to occur under low light and during photosynthetic induction (Kromdijk et al., 2010; Medeiros et al., 2022); the resulting photorespiratory intermediates could act as a carbon reservoir from which to build C_3_ and C_4_ metabolite pools (Fu and Walker, 2022). The presence of an endogenous source of carbon is supported by the inability to account for the net increase in C_3_ and C_4_ cycle intermediates during light induction based on rates of CO_2_ assimilation alone (Leegood and Furbank, 1984; Usuda, 1985).

Whilst the experimental evidence and putative mechanisms detailed above may indeed suggest that C_4_ species could be more affected by transient decreases in photosynthetic efficiency during induction relative to steady state than C_3_ species, most of the work does not directly compare C_3_ and C_4_ species in a common experiment, but instead often focuses on a single species, such as maize (Zelitch et al., 2009; Kromdijk et al., 2010; Medeiros et al., 2022). Some direct comparisons between C_3_ and C_4_ photosynthesis have been made in sets of contrasting grass species (Lee et al., 2022) and species within the same genus (Kubásek et al., 2013), but so far the only C_3_ and C_4_ species studied that share a relatively recent common ancestor are *Flaveria* (Li et al., 2021). Phylogenetic distance can strongly confound the apparent photosynthetic differences observed (Taylor et al., 2010) and to confirm whether observed differences are due to photosynthetic pathway or evolutionary variation, studies conducted on phylogenetically linked C_3_ and C_4_ species are necessary. Furthermore, despite striking similarities in the anatomy and biochemistry of C_4_ species from diverse evolutionary origins, there is still great diversity amongst species. Some common variations are the main decarboxylases utilised to release CO_2_ in BS cells: nicotinamide adenine dinucleotide-malic enzyme (NAD-ME), nicotinamide adenine dinucleotide phosphate-malic enzyme (NADP-ME), and phosphoenolpyruvate carboxykinase (PEPCK). Although C_4_ subtypes had initially been defined by these main decarboxylases (Hatch et al., 1975), more recent work suggests there is a greater degree of nuance than traditional C_4_ classifications connote, as NADP-ME and NAD-ME often operate alongside a PEPCK auxiliary pathway, and the energetic requirements of C_4_ photosynthesis render a pure PEPCK subtype unlikely (Wang et al., 2014a). Variations across C_4_ species and phylogenetic distance are thus important considerations when trying to derive generic differences between C_3_ and C_4_ photosynthesis.

In this study we analysed steady state photosynthesis and photosynthetic induction in three phylogenetically linked pairs of C_3_ and C_4_ species from *Alloteropsis*, *Flaveria*, and *Cleome* genera, representative of monocots and dicots, and all three C_4_ decarboxylation enzymes. Photosynthetic gas exchange was measured in response to a step-change to moderate and strongly saturating light intensities to characterise differences in photosynthetic induction rates. Experiments were conducted at both 21% and 2% O_2_ concentration to evaluate the role of photorespiration during induction. Activation of CO_2_ assimilation at the start of light induction was slower in all C_4_ species compared to their C_3_ counterparts although the mechanism of impairment varied across genera. Furthermore, although both C_3_ and C_4_ *Flaveria* had greater CO_2_ assimilation under 2% O_2_, assimilation in C_3_ and C_4_ *Alloteropsis* species as well as C_3_ *T. hassleriana* was negatively impacted by low O_2_. The variation in responses highlights the natural diversity of C_4_ species, and the importance of controlling for phylogenetic distance in comparisons between C_3_ and C_4_ photosynthesis.

## Materials and methods

### Plant materials

**Figure 1:**
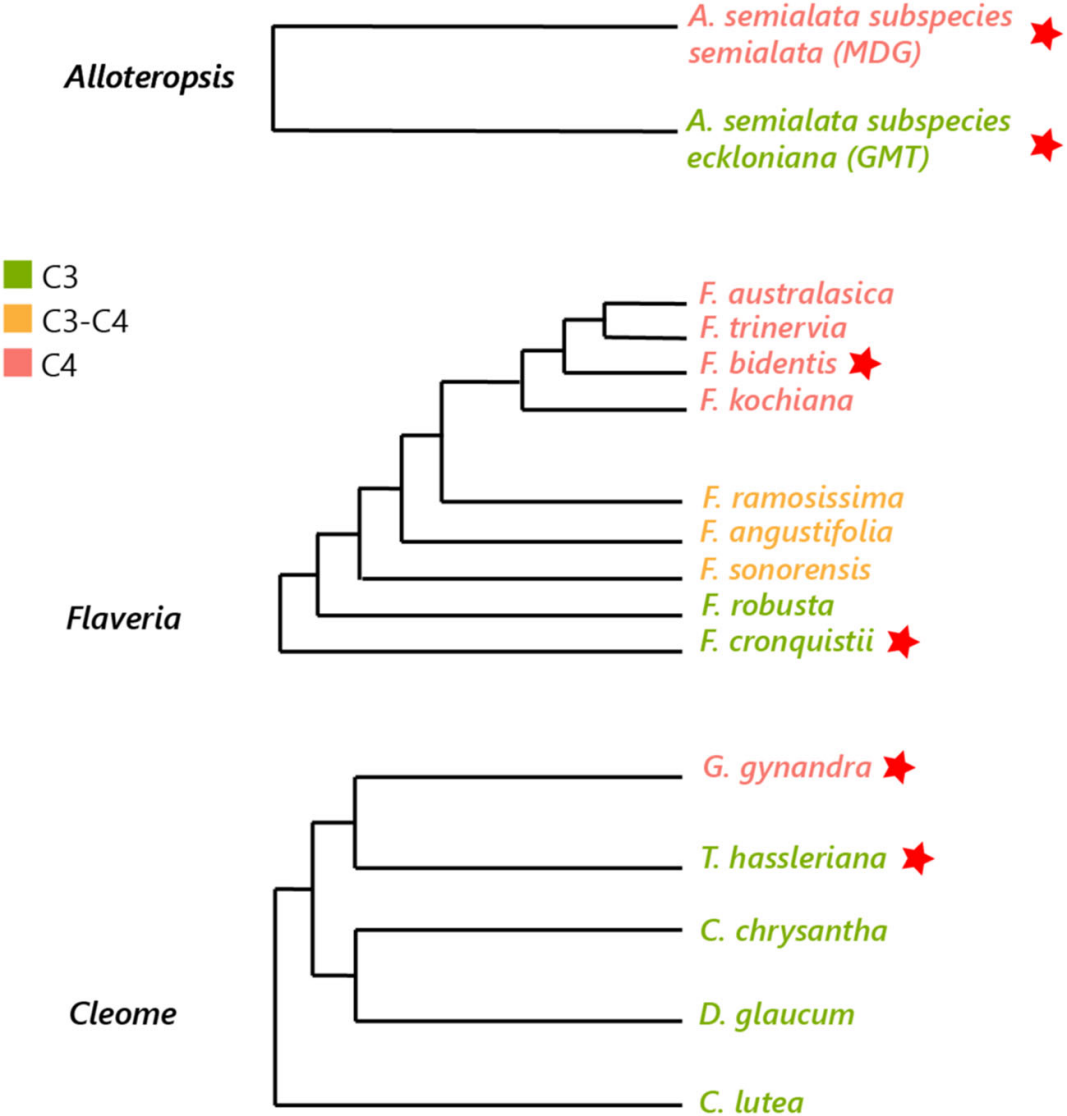
Phylogenetic tree of *Alloteropsis*, *Flaveria* and *Cleome* genera. Species used in the study are marked with a red star (adapted from Ibrahim et al., 2009; Lyu et al., 2015; Shi et al., 2021).

Three pairs of phylogenetically linked *Alloteropsis*, *Flaveria* and *Cleome* C_3_ and C_4_ species were selected to decrease evolutionary variation within each pair (see **Figure 1**) (Lyu et al., 2015) but maintain significant evolutionary distance between the three genera, as C_4_ origins arose ~ 17 million years ago (Ma) in *Cleome*, ~ 2 Ma in *Flaveria*, and even earlier in *Alloteropsis* (Christin et al., 2011; Lundgren et al., 2015). The selected species include monocots (C_3_ *Alloteropsis semialata* subspecies *semialata* accession *GMT* and C_4_ *Alloteropsis semialata* subspecies *eckloniana* accession *MDG*), dicots (C_3_ *Flaveria cronquistii*, C_4_ *Flaveria bidentis*, C_3_ *Tarenaya hassleriana* and C_4_ *Gynandropsis gynandra*), and the three major decarboxylase enzymes of the C_4_ pathway, which have been suggested to be dominant in different C_4_ species: PEPCK in mixed NADP-ME-PEPCK pathway in *A. semialata MDG* (Ueno and Sentoku, 2006), NADP-ME in *F. bidentis* (Gowik et al., 2011), and NAD-ME in *G. gynandra* (Bräutigam et al., 2010).

### Plant growth and propagation

All plants were measured during the vegetative growth phase. Plants were grown in Levington Advance M3 compost (Scotts, Ipswich, UK) mixed with Miracle-Gro All Purpose Continuous Release Osmocote (Scotts Miracle-Gro Company, Marysville, OH, USA; 4 L compost: 25g Osmocote). Medium vermiculite was added to the *Alloteropsis* soil mix (4 L compost: 1 L vermiculite: 25g Osmocote) to prevent waterlogging.

The *Alloteropsis GMT* and *MDG* accessions were vegetatively propagated and grown on 2 L pots under well-watered conditions in a glasshouse at 18-25°C, 40-60% relative humidity (RH), with supplemental lightning provided to ensure at least 140-160 µmol m^-2^ s^-1^ photon flux density (PFD) across a 16-hour photoperiod. Plants were measured two weeks after propagation.

The *Flaveria* and *Cleome* species were grown under well-watered conditions in a Conviron growth room (Conviron Ltd., Winnipeg, MB, CA) at 20°C temperature, 60% RH, and 150 µmol m^-2^ s^-1^ PFD over a 16-hour photoperiod. Because *F. cronquistii* requires vegetative propagation, plants from both *Flaveria* species were propagated from lateral shoot cuttings – *F. bidentis* plants were initially grown from seed and then propagated. Cuttings were dipped in Doff Hormone Rooting Powder (Doff Portland Ltd., Hucknall, UK) to induce root development, and *Flaveria* cuttings were grown on 0.25 L pots and measured after 8-10 weeks.

*Cleome* germination was induced under sterile conditions at 30°C/20°C day/night cycle for *T. hassleriana*, and at 30°C for *G. gynandra*. Germinated seeds were initially sown in 24-cell seed trays before transfer to 0.25 L pots. As *G. gynandra* has a lower development rate than *T. hassleriana*, germination was staggered so both species could be measured at approximately the same developmental stage, after 8-10 weeks for *G. gynandra* and 4-6 weeks for *T. hassleriana*.

### Gas exchange and chlorophyll fluorescence

Gas exchange and chlorophyll fluorescence were measured simultaneously using an open gas exchange system (LI-6400XT, LI-COR, Lincoln, NE, USA) with an integrated leaf chamber fluorometer (6400-40 LCF). Leaves were measured in a 2 cm^2^ chamber at 25°C block temperature, 410 ppm sample CO_2_ concentration, and 50-65% RH with flow of 300 µmol s^-1^. Average leaf VPD was 1.1 ± 0.1 kPa at the start and 1.35 ± 0.1 kPa at the end of the light treatment. Actinic light was provided by the LCF and composed of 10% blue (470 nm) and 90% red light (630 nm).

The LCF used a 0.25 Hz modulated measuring light and a multiphase flash (Loriaux et al., 2013) to measure steady (*F’*) and maximal (*F_m_’*) fluorescence to derive the quantum yield of Photosystem II (ΦPSII) (Genty et al., 1989).

For experiments using 2% O_2_, a pre-mixed 2% O_2_ and 98% N_2_ gas mixture was supplied to the LI-6400XT through the air inlet, using a mass flow controller (EL-FLOW, Bronkhorst High-tech BV, Ruurlo, NL) and an open T-junction to regulate constant surplus flow. Infrared Gas Analyzer (IRGA) calibration was adjusted to the O_2_ gaseous composition in the instrument settings prior to measurement.

### Leaf absorptance

Light absorptance of the plants used in experiments was measured with an integrating sphere (LI-1800-12, LI-COR) optically connected to a miniature spectrometer (Li-Cor, 1988). Incident PFD was converted to absorbed photon flux density (PFD_abs_) using the measured leaf absorptance of the emission wavelengths of the 6400-40 LCF light source. The specific absorptance values can be found in **Supplementary Material 1**.

### Steady state light response curves

Steady state light response curves of photosynthetic gas exchange were measured for all species at both 21% and 2% O_2_. Leaves were light-adapted at 1000 µmol m^-2^ s^-1^ PFD and once CO_2_ assimilation and stomatal conductance reached steady state, gas exchange and chlorophyll fluorescence parameters were measured in a descending gradient of light intensity: 2000, 1700, 1500, 1200, 1000, 800, 600, 400, 300, 200, 100, 75, 30, and 0 µmol m^-2^ s^-1^ PFD. Gas exchange and chlorophyll fluorescence parameters were logged after 120 – 240 s, when leaf intracellular CO_2_ concentration (C_i_) and CO_2_ assimilation were stable.

To analyse steady state responses, a non-rectangular hyperbola was fitted to the light response curves (Stinziano et al., 2021). The quantum yield of assimilation (α) was derived from the initial slope, and the light-saturated photosynthetic rate (A_max_) from the asymptote of the curve. C_i_ values obtained above 600 µmol m^-2^ s^-1^ PFD were averaged to estimate light-saturated C_i_ (C_i max_).

Approximate light intensities at the inflection point (600 µmol m^-2^ s^-1^ PFD) and in the saturating part of the response (1500 µmol m^-2^ s^-1^ PFD) were used in the light induction experiments. Respiration in the light (*Rd*) was estimated for all species at each O_2_ concentration as the y-intercept using a linear regression of the initial light response curve slope. To account for the Kok effect, measurements in darkness and at 30 µmol m^-2^ s^-1^ light intensity were not included in the regression (Kok, 1949).

### Light induction experiments and analysis of lag in carbon assimilation

Leaves were dark-adapted until stomatal conductance reached constant levels (between 30-60 minutes depending on the species), illuminated with 600 µmol m^-2^ s^-1^ or 1500 µmol m^-2^ s^-1^ PFD for 1 hour, and then returned to darkness for another half hour. Starting from the last 5 minutes of initial dark adaption, gas exchange parameters were logged every minute, and chlorophyll fluorescence parameters at 5, 15, 25, 35, 45, and 60 minutes after starting light exposure. Light induction experiments at both light intensities were conducted at 21% and 2% O_2_. Carbon assimilation was corrected for respiration to determine net photosynthetic CO_2_ assimilation (A_CO2_) using the *Rd* obtained from light response curves.

To analyse photosynthetic responses across the induction period, the trapezoidal rule (Jawień, 2014) was used to integrate the area under the curve (AUC) (Makowski et al., 2019) of A_CO2_ during the 0 – 5, 5 – 10, and 10 – 60 minute phases of light exposure.

### Alternative electron sinks

The electron cost of assimilation can be approximated by the ΦPSII/ΦCO_2_ ratio (Genty et al., 1989; Oberhuber and Edwards, 1993), with ΦCO_2_ being the quantum yield of CO_2_ assimilation (**Equation 1**). Lower ratios are associated with greater coupling as more electrons captured by PSII go towards CO_2_ assimilation (Krall and Edwards, 1990). For light response curves the ΦPSII/ΦCO_2_ ratio was calculated for the values obtained at 600 µmol m^-2^ s^-1^ PFD (*600* ΦPSII/ΦCO_2_) and 1500 µmol m^-2^ s^-1^ PFD (*1500* ΦPSII/ΦCO_2_). During light induction ΦPSII/ΦCO_2_ values were taken from across the light period at each intensity. Data points were excluded if calculated ΦCO_2_ showed negative values.


Equation 1 $$\Phi CO_{2}=\frac{\left(A_{CO2}+Rd\right)}{PFD_{abs}}$$

### Statistical analysis

All statistical analyses were conducted separately on paired *Alloteropsis, Flaveria*, and *Cleome* light response curves, light induction at 600 µmol m^-2^ s^-1^ PFD, and light induction at 1500 µmol m^-2^ s^-1^ PFD. Mean and standard error of the mean of light response curve parameters (A_max_, α, C_i max_, *600* ΦPSII/ΦCO_2_ and *1500* ΦPSII/ΦCO_2_), A_CO2_ AUC at different phases of induction, and ΦPSII/ΦCO_2_ across light induction were calculated. Linear mixed models (LMMs) were fitted to the light response curve parameters and A_CO2_ AUC at different phases of induction using photosynthetic pathway, O_2_ concentration and their interaction as fixed effects; and to ΦPSII/ΦCO_2_ across light induction using photosynthetic pathway, O_2_ concentration, time, and their interactions as fixed effects. Time of day and measured plant were included as random effects in all models. Two and three-way ANOVA tables for the fixed effects were generated from the LMMs using the Satterthwaite’s approximation method (Kuznetsova et al., 2017). The data was independent and assumptions of normality, homogeneity of variance and sphericity were satisfied.

All data analysis and plot generation was done on RStudio 1.3 (RStudio Team, 2022) with R 4.1.1 (R Core Team, 2021) using the tidyverse (Wickham et al., 2019), RColorBrewer (Neuwirth, 2014), lme4 (Bates et al., 2015), lmerTest (Kuznetsova et al., 2017) and bayestestR libraries (Makowski et al., 2019).

## Results

### Steady state measurements confirm canonical differences in CO_2_ assimilation between C_3_ and C_4_ species

**Figure 2:**
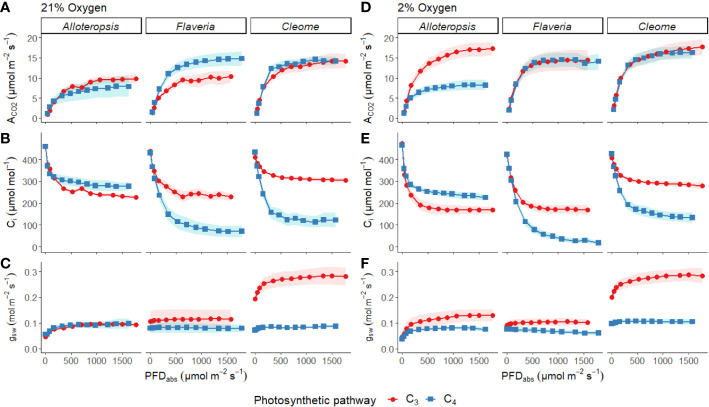
Measurements of gas exchange traits during light response curves for phylogenetically linked C_3_ and C_4_ *Alloteropsis*, *Flaveria* and *Cleome* species under 21% and 2% O_2_. Plots show net CO_2_ assimilation (A_CO2_, **A, D**), intercellular CO_2_ concentration (C_i_, **B, E**), and stomatal conductance to water vapour (g_sw_, **C, F**) as a function of absorbed light intensity. Ribbons represent standard error of the mean (n=5).

**Figure 3:**
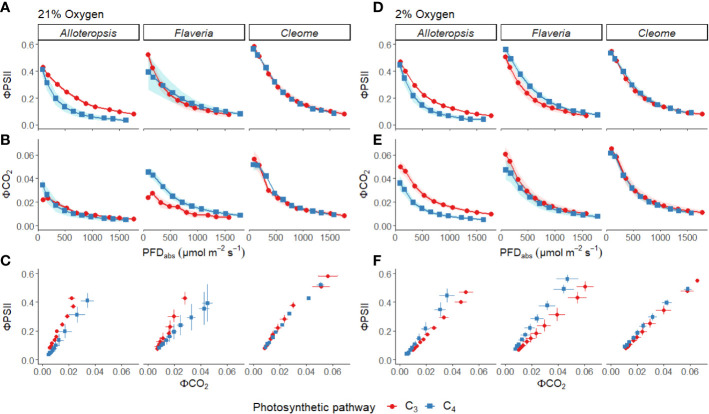
Measurements of gas exchange and chlorophyll fluorescence traits during light response curves for phylogenetically linked C_3_ and C_4_ *Alloteropsis*, *Flaveria* and *Cleome* species under 21% and 2% O_2_. Plots show quantum yield of PSII (ΦPSII, **A**, **D**), and quantum yield of CO_2_ assimilation (ΦCO_2_, **B, E**) as a function of absorbed light intensity. Ribbons represent standard error of the mean (n=5). Plots **(C, F)** display the relationship between ΦPSII and ΦCO_2_. Error bars represent standard error of the mean for both parameters.

Light response curves were used to first characterise C_3_ and C_4_ responses under steady state at 21% (**Figures 2A-C**, **3A-C** and **Table 1**). The responses of C_4_ species in comparison to their C_3_ phylogenetic pairs were genus specific – C_4_ *F. bidentis* had higher maximum rates of net carbon assimilation (A_max_) than C_3_ *F. cronquistii* (*P* = 0.03; C_3_ 12.5 ± 1.3 vs C_4_ 16.7 ± 1.0 µmol m^-2^ s^-1^), but A_max_ values in C_4_ *G. gynandra* were similar to those found in C_3_ *T. hassleriana* (*P* = 0.24; C_3_ 15.5 ± 2.0 vs C_4_ 16.7 ± 0.7 µmol m^-2^ s^-1^), and A_max_ values in C_4_ *A. semialata MDG* also were similar to C_3_ *A. semialata GMT* (*P* = 0.02; C_3_ 11.8 ± 1.1 vs C_4_ 10.2 ± 2.7 µmol m^-2^ s^-1^). A two-way ANOVA (**Table 2**) showed photosynthetic pathway had a significant effect on C_i_ during light saturation in all genera. However, whilst the C_4_ pathway was associated with lower C_i max_ in *Flaveria* (*P* ≤ 0.001; C_3_ 238 ± 19 vs C_4_ 86 ± 27 µmol mol^-1^) and *Cleome* (*P* ≤ 0.001; C_3_ 310 ± 8 vs C_4_ 125 ± 27 µmol mol^-1^), in *Alloteropsis* the C_4_ association was instead with higher C_i max_ (*P* ≤ 0.01; C_3_ 242 ± 9 vs C_4_ 285 ± 21 µmol mol^-1^). **Figure 2C** shows that the lower C_i_ of C_4_ species corresponded to lower stomatal conductance, excepting C_4_ *A. semialata MDG*, where stomatal conductance to water vapour (g_sw_) was similar to C_3_ *A. semialata GMT* across the light response.

**Table 1:** Light response curve parameters estimated from steady state light response curves under 21% and 2% O_2_ on phylogenetically linked C_3_ and C_4_ *Alloteropsis*, *Flaveria* and *Cleome* species.

		21% Oxygen		2% Oxygen	
Genus	Parameter	C_3_	C_4_	C_3_	C_4_
*Alloteropsis*	A_max_ (µmol m^-2^ s^-1^)	11.8 ± 1.1	10.2 ± 2.7	20.9 ± 2.4	10.3 ± 1.4
	α	0.04 ± 0.00	0.10 ± 0.02	0.08 ± 0.01	0.09 ± 0.01
	C_i max_ (µmol mol^-1^)	242 ± 9	285 ± 21	171 ± 19	240 ± 14
	*600* ΦPSII/ΦCO_2_	16.7 ± 1.8	11.3 ± 2.4	8.9 ± 1.7	10.0 ± 2.4
	*1500* ΦPSII/ΦCO_2_	16.2 ± 1.6	8.6 ± 2.1	7.5 ± 1.2	6.8 ± 2.6
	*Rd*	0.9 ± 0.2	1.4 ± 0.6	1.5 ± 0.3	1.5 ± 0.1
*Flaveria*	A_max_ (µmol m^-2^ s^-1^)	12.5 ± 1.3	16.7 ± 1.0	15.6 ± 1.7	15.3 ± 1.8
	α	0.07 ± 0.02	0.06 ± 0.01	0.07 ± 0.01	0.06 ± 0.01
	C_i max_ (µmol mol^-1^)	238 ± 19	86 ± 27	174 ± 18	43 ± 15
	*600* ΦPSII/ΦCO_2_	14.0 ± 0.3	9.0 ± 5.2	7.6 ± 0.3	12.6 ± 3.2
	*1500* ΦPSII/ΦCO_2_	12.2 ± 0.6	9.5 ± 2.2	8.1 ± 0.6	11.1 ± 3.2
	*Rd*	0.9 ± 0.3	1.0 ± 0.4	0.6 ± 0.2	0.4 ± 0.2
*Cleome*	A_max_	15.5 ± 2.0	16.7 ± 0.7	20.9 ± 2.5	17.7 ± 1.5
	α	0.07 ± 0.01	0.09 ± 0.02	0.15 ± 0.03	0.08 ± 0.01
	C_i max_ (µmol mol^-1^)	310 ± 8	125 ± 27	290 ± 11	149 ± 21
	*600* ΦPSII/ΦCO_2_	12.3 ± 0.8	11.7 ± 1.1	7.7 ± 1.0	9.4 ± 0.5
	*1500* ΦPSII/ΦCO_2_	10.5 ± 0.5	9.8 ± 1.0	8.7 ± 0.7	8.8 ± 0.3
	Rd	0.3 ± 0.2	1.8 ± 0.7	2.1 ± 0.7	0.8 ± 0.5

The light-saturated photosynthetic rate (A_max_) and the quantum yield of assimilation (α) were calculated by fitting the light response curves with a non-rectangular hyperbola. The C_i_ at light saturation point (C_i max_) is the average C_i_ at PFD **≥** 600 µmol m^-2^ s^-1^. Values for *600* ΦPSII/ΦCO_2_ and *1500* ΦPSII/ΦCO_2_ were taken at PFD = 600 µmol m^-2^ s^-1^ and PFD =1500 µmol m^-2^ s^-1^. Means and standard error of the mean are shown (n = 5).

**Table 2:** ANOVA table of modelled light response curve parameters for phylogenetically linked C_3_ and C_4_ *Alloteropsis*, *Flaveria* and *Cleome* species.

		*Alloteropsis*			*Flaveria*			*Cleome*		
Parameter		PP	[O_2_]	PP:[O_2_]	PP	[O_2_]	PP:[O_2_]	PP	[O_2_]	PP:[O_2_]
A_max_	df;*F*-value;*P*-value	1,15; 7.40; **0.02**	1,15; 4.47; 0.06	1,15; 5.90; **0.03**	1,15; 5.81; **0.03**	1,15; 1.40; 0.26	1,15; 7.73; **0.01**	1,15; 0.29; 0.59	1,15; 3.19; 0.09	1,15; 1.51; 0.24
α	df;*F*-value;*P*-value	1,15; 3.89; 0.06	1,15; 1.85; 0.20	1,15; 3.69; 0.07	1,15; 1.38; 0.26	1,15; 0.03; 0.87	1,15; 0.26; 0.62	1,15; 1.45; 0.24	1,15; 3.77; 0.07	1,15; 5.58; **0.03**
C_imax_	df;*F*-value;*P*-value	1, 16; 11.06; **≤0.01**	1, 16; 12.00; **≤0.01**	1, 16; 0.54; 0.45	1, 16; 49.07; **≤0.001**	1, 16; 6.75; **0.02**	1, 16; 0.27; 0.61	1, 16; 70.23; **≤0.001**	1, 16; 0.00; 0.93	1, 16; 1.27; 0.27
*600* ΦPSII/ΦCO_2_	df;*F*-value;*P*-value	1,16; 6.05; **0.03**	1,16; 24.29; **≤0.001**	1,16; 12.28; **≤0.01**	1,16; 2.01; 0.16	1,16; 5.31; **0.05**	1,16; 5.95; **0.03**	1,16; 0.05; 0.84	1,16; 30.01; **≤0.01**	1,16; 5.39; **0.05**
*1500* ΦPSII/ΦCO_2_	df;*F*-value;*P*-value	1,16; 22.75; **≤0.001**	1,16; 35.56; **≤0.001**	1,16; 15.67; **≤0.01**	1,16; 0.37; 0.56	1,16; 1.55; 0.26	1,16; 6.68; **0.05**	1,16; 0.08; 0.78	1,16; 15.61; **0.01**	1,16; 5.57; **0.05**

Photosynthetic pathway, PP. O_2_ concentration, [O_2_]. Interaction effect, PP:[O_2_]. Table shows degrees of freedom; *F*-value; and *P*-value. Significant (*a<* 0.05) *P*-values are shown in bold.

ΦPSII decreased exponentially with higher light intensities. Although ΦPSII values were very similar across C_3_ and C_4_ pairs in *Flaveria* and *Cleome*, more pronounced decreases were observed in C_4_ *A. semialata MDG* than in C_3_ *A. semialata GMT* (**Figure 3A**). ΦCO_2_ was also lower at higher light intensities, following a similar pattern to A_max_ across the light response, as ΦCO_2_ was similar between C_3_ and C_4_ species in *Alloteropsis* and *Cleome*, but higher in C_4_ *F. bidentis* compared to C_3_ *F. cronquistii* (**Figure 3B**). These differences across genera were also apparent for the observed ΦPSII and ΦCO_2_ ratios, but not always significantly so. C_4_ *F. bidentis* had lower ΦPSII/ΦCO_2_ than C_3_ *F. cronquistii* (*P* = 0.16; C_3_ 14.0 ± 0.3 vs C_4_ 9.0 ± 2.4 at PFD = 600 µmol m^-2^ s^-1^, and *P* = 0.56; C_3_ 12.2 ± 0.6, C_4_ 9.5 ± 2.2 at PFD = 1500 µmol m^-2^ s^-1^) and the same was observed for C_4_ *A. semialata MDG* compared to C_3_ *A. semialata GMT* (*P* = 0.03; C_3_ 16.7 ± 1.8 vs C_4_ 11.3 ± 2.4 at PFD = 600 µmol m^-2^ s^-1^, and *P* ≤ 0.001; C_3_ 16.2 ± 1.6 vs C_4_ 8.6 ± 2.1 at PFD = 1500 µmol m^-2^ s^-1^), suggesting that in these C_4_ species less electron transfer through PSII is needed per CO_2_ fixed. **Figure 3C** shows that the lower ratio of ΦPSII to ΦCO_2_ in C_4_ *Flaveria* and *Alloteropsis* in relation to their C_3_ counterparts was observed across most light intensities, although the difference appeared to be marginal at higher light intensities. At 21% O_2_, both C_3_ and C_4_ *Cleome* species had very similar ΦPSII/ΦCO_2_ (*P* = 0.84; C_3_ 12.3 ± 0.8 vs C_4_ 11.7 ± 1.1 at PFD = 600 µmol m^-2^ s^-1^, and *P* = 0.78; C_3_ 10.5 ± 0.5 vs C_4_ 9.8 ± 1.0 at PFD = 1500 µmol m^-2^ s^-1^).

Light response curves were also performed at 2% O_2_ to minimize photorespiration (**Figures 2D-F**, **3D-F** and **Table 1**). All three C_3_ species had substantially higher CO_2_ assimilation rates under low O_2_ – A_max_ was around 75% higher in C_3_ *A. semialata MDG*, 25% higher in C_3_ *F. cronquistii*, and 35% higher in C_3_ *T. hassleriana* than under 21% O_2_. 2% O_2_ also led to a decrease in C_i max_ in *Alloteropsis* (*P* ≤ 0.01 in C_3_ 171 ± 19 vs C_4_ 240 ± 14 µmol mol^-1^) and *Flaveria* (*P* = 0.02; C_3_ 174 ± 18 vs C_4_ 43 ± 15 µmol mol^-1^) but no significant change in either *Cleome* species (*P* = 0.93; C_3_ 290 ± 11 vs C_4_ 149 ± 21 µmol mol^-1^), where A_CO2_ and g_sw_ appeared tightly coordinated. The increase in A_max_ was not mirrored in C_4_ species, as evidenced by significant interactions (**Table 2**) between photosynthetic pathway and oxygen in *Alloteropsis* (*P* = 0.03) and *Flaveria* (*P* = 0.01), where low O_2_ concentrations were associated with higher A_max_ on C_3_ but not C_4_ species. (**Table 2**). Similar patterns were observed for the two *Cleome* species, however the increase in A_max_ at 2% O_2_ for C_3_ *T. hassleriana* was less pronounced than for the other C_3_ species and instead the initial slope α appeared to be subject to a significant interaction between effects of photosynthetic pathway and O_2_ (*P* = 0.03). The different effects on assimilation at low O_2_ between C_3_ and C_4_ species were also reflected in the changing relationship between ΦPSII and ΦCO_2_ (**Figures 3C, F**) – photosynthetic pathway and O_2_ concentration were found to have significant interactions on ΦPSII/ΦCO_2_ in all three genera (**Table 2**, *P* = 0.05 in *Flaveria* and *Cleome*, *P* ≤ 0.01 in *Alloteropsis*), due to decreases in ΦPSII/ΦCO_2_ in C_3_ species at 2% O_2_ not observed in C_4_ species. This data confirms that photorespiration is a significant electron sink under steady state for all three C_3_ species, whereas the steady state suppression of photorespiration at 21% O_2_ in the C_4_ species is sufficient to prevent any significant further decreases in ΦPSII/ΦCO_2_ under 2% O_2_.

### Substantial differences in photosynthetic traits exist between C_3_ and C_4_ species during light induction

**Figure 4:**
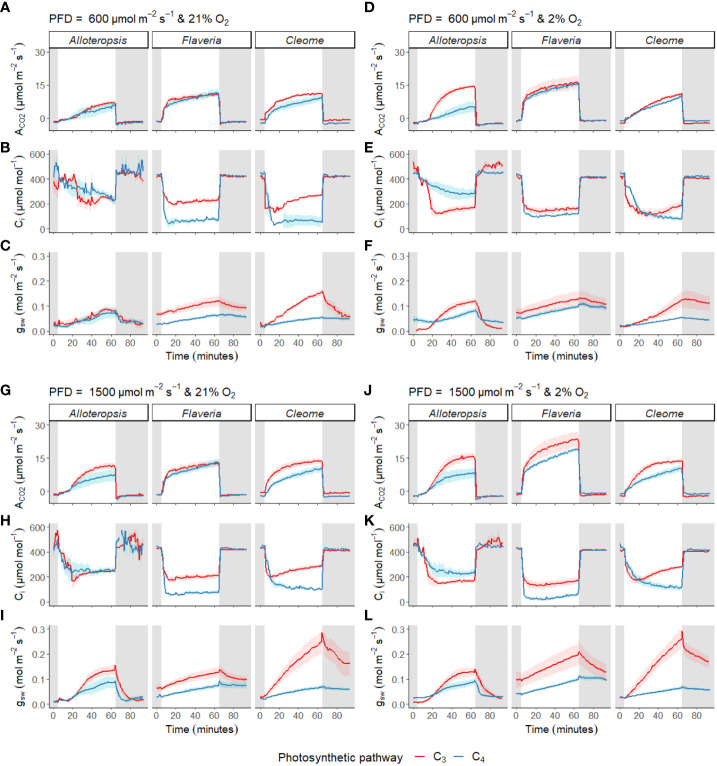
Measurements of gas exchange during light induction in phylogenetically linked C_3_ and C_4_ *Alloteropsis*, *Flaveria* and *Cleome* species. Leaves were acclimated to darkness, exposed to 1 hour of light, and returned to darkness for another half hour. Plots show net CO_2_ assimilation (A_CO2_, **A, D, G, J**), intercellular CO_2_ concentration (C_i_, **B, E, H, K**) and stomatal conductance to water vapour (g_sw_, **C, F, I, L**) across the light induction experiment, at PFD = 600 µmol m^-2^ s^-1^ and PFD = 1500 µmol m^-2^ s^-1^, in 21% and 2% O_2_. Ribbons represent standard error of the mean (n = 5).

Photosynthetic induction rates were measured in leaves exposed to 600 µmol m^-2^ s^-1^ or 1500 µmol m^-2^ s^-1^ PFD from darkness (**Figures 4A-C, G-I**). The light induction response across all species and light intensities generally consisted of gradual stomatal opening in line with a rise in A_CO2_ towards steady state, and a sharp drop in C_i_ at the start of induction, followed by a gradual recovery.

In addition to these general patterns, several differences were observed across genera and between paired species. Stomata tended to open more quickly in the C_3_ species than in their respective C_4_ counterparts. Furthermore, the speed of A_CO2_ induction appeared to vary between some C_3_ and C_4_ pairs; where carbon assimilation was notably slower to induce in C_4_ *G. gynandra* compared to C_3_ *T. hassleriana* under both light intensities, with more subtle differences observed in the *Alloteropsis* and *Flaveria* C_3_ and C_4_ pairs.

#### Reductions in assimilation of CO_2_ at the start of induction in C_3_ and C_4_ species vary across genera

**Figure 5:**
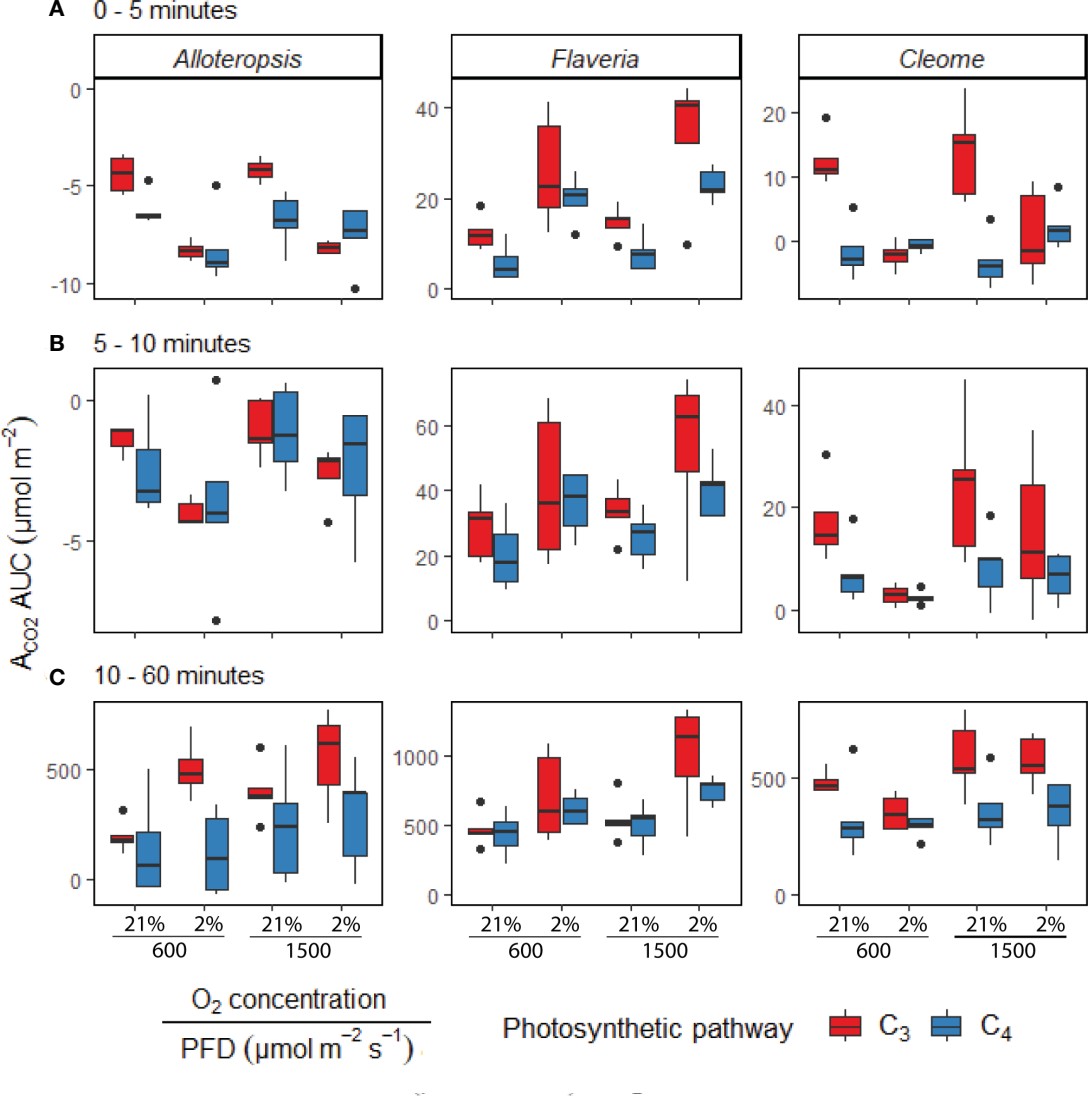
Boxplots of cumulative CO_2_ assimilation over different phases of light induction in phylogenetically linked C_3_ and C_4_ *Alloteropsis*, *Flaveria* and *Cleome* species, under different light and O_2_ treatments (n = 5 for each combination of species/measurement condition). Box edges represent first and third quartiles, the solid line indicates the median, and points represent outliers beyond 1.5 times the interquartile range. The area under the curve (AUC) was calculated from the A_CO2_ of light induction experiments where plants at 21% or 2% O_2_ concentrations were dark-adapted and exposed to PFD = 600 µmol m^-2^ s^-1^ or PFD = 1500 µmol m^-2^ s^-1^ for 1 hour. Plots show the AUC of induction during 0 – 5 minutes **(A)**, 5 – 10 minutes **(B)**, and 10 – 60 minutes **(C)**. Two-way ANOVAs (**Table 3**) were used to test the effect of photosynthetic pathway, O_2_ concentration and their interaction on A_CO2_ AUC at different phases of light induction in *Alloteropsis, Flaveria*, and *Cleome*.

In order to systematically explore C_3_ and C_4_ differences in the activation of CO_2_ assimilation, the induction time-series were subdivided into three periods, 0 – 5 min, 5 – 10 min, and the remaining 10 – 60 min, and integrated carbon assimilation (AUC) was calculated for each period (**Figure 5**). During the 0 – 5 min period, C_4_ *F. bidentis* had lower AUC than C_3_ *F. cronquistii* under both PFD = 600 µmol m^-2^ s^-1^ (*P* = 0.09; C_3_ 12.4 ± 1.7 vs C_4_ 5.8 ± 2.0 µmol m^-2^) and PFD = 1500 µmol m^-2^ s^-1^ (*P* = 0.02; C_3_ 14.8 ± 1.8 vs C_4_ 8.0 ± 1.8 µmol m^-2^), with the difference becoming significant under higher light (**Table 3**). The difference in assimilated CO_2_ between C_3_ and C_4_ species at the start of induction was even more pronounced in *Cleome*, where the AUC of C_4_ *G. gynandra* was significantly lower than that of C_3_ *T. hassleriana* under both light intensities (*P* ≤ 0.01; C_3_ 12.6 ± 2.0 vs C_4_ -1.4 ± 1.9 µmol m^-2^ at PFD = 600 µmol m^-2^ s^-1^, and *P* ≤ 0.01; C_3_ 13.8 ± 3.2 vs C_4_ -3.1 ± 1.8 µmol m^-2^ at PFD = 1500 µmol m^-2^ s^-1^). The AUC in C_4_ *G. gynandra* continued to be significantly lower than in C_3_ *T. hassleriana* under both light intensities during the following two periods of induction analysed (**Table 3**). In contrast, the significant difference in cumulative CO_2_ uptake between *Flaveria* species was only significant during the first five minutes of induction (**Figure 5A**). Thus, there was a more pronounced lag in CO_2_ assimilation during induction in C_4_ photosynthesis in *Flaveria* and *Cleome* than in C_3_ photosynthesis in the same genera. This was especially apparent in relation to the steady state comparison between both species-pairs (**Figure 2A**). In *Alloteropsis*, the C_4_ *A. semialata MDG* also started at lower AUC than C_3_ *A. semialata GMT* during the 0 – 5 min period under both light intensities (*P* = 0.36; C_3_ -4.4 ± 0.5 vs C_4_ -6.22 ± 0.39 at PFD = 600 µmol m^-2^ s^-1^, and *P* = 0.07; C_3_ -4.2 ± 0.2, vs C_4_ -6.8 ± 0.6 at PFD = 1500 µmol m^-2^ s^-1^), but the difference in AUC between pathways was only found to be significant for the final 10 – 60 min (*P* ≤ 0.01; C_3_ 194.8 ± 34.3 vs C_4_ 142.2 ± 101.0 at PFD = 600 µmol m^-2^ s^-1^, and *P* = 0.03; C_3_ 403.3 ± 59.5 vs C_4_ 241.5 ± 115.6 at PFD = 1500 µmol m^-2^ s^-1^).

**Table 3:** ANOVA table of the carbon assimilation AUC of different phases of light induction for phylogenetically linked C_3_ and C_4_ *Alloteropsis*, *Flaveria* and *Cleome* species.

			*Alloteropsis*			*Flaveria*			*Cleome*		
Light intensity (µmol m^-2^ s^-1^)	Induction phase (minutes)		PP	[O_2_]	PP:[O_2_]	PP	[O_2_]	PP:[O_2_]	PP	[O_2_]	PP:[O_2_]
600	0 – 5	df;*F*-value;*P*-value	1,16; 1.34; 0.26	1,16; 27.85; **≤0.001**	1,16; 3.13; 0.10	1,16; 3.18; 0.09	1,16; 15.08; **≤0.01**	1,16; 0.00; 0.95	1,16; 15.56; **≤0.01**	1,16; 15.12; **≤0.01**	1,16; 25.03; **≤0.001**
	5 – 10	df;*F*-value;*P*-value	1,16; 0.20; 0.66	1,16; 5.81; **0.02**	1,16; 0.80; 0.38	1,16; 0.86; 0.36	1,16; 3.60; 0.08	1,16; 0.05; 0.83	1,16; 5.01; **0.04**	1,16; 10.06; **≤0.01**	1,16; 2.95; 0.11
	10 – 60	df;*F*-value;*P*-value	1,16; 8.87; **≤0.01**	1,16; 3.68; 0.07	1,16; 5.14; **0.03**	1,16; 0.39; 0.54	1,16; 4.46; **0.05**	1,16; 0.08; 0.78	1,16; 4.58; **0.05**	1,16; 2.41; 0.14	1,16; 0.89; 0.36
1500	0 – 5	df;*F*-value;*P*-value	1,16; 3.81; 0.07	1,16; 22.30; **≤0.001**	1,16; 10.14; **≤0.01**	1,16; 6.39; **0.02**	1,16; 24.21; **≤0.001**	1,16; 0.33; 0.57	1,16; 9.48; **≤0.01**	1,16; 2.07; 0.17	1,16; 12.85; **≤0.01**
	5 – 10	df;*F*-value;*P*-value	1,16; 0.00; 0.99	1,16; 3.40; 0.08	1,16; 0.07; 0.78	1,16; 2.54; 0.13	1,16; 7.06; **0.01**	1,16; 0.12; 0.73	1,16; 5.90; **0.02**	1,16; 1.18; 0.29	1,16; 0.49; 0.49
	10 – 60	df;*F*-value;*P*-value	1,16; 5.09; **0.03**	1,16; 1.06; 0.31	1,16; 0.33; 0.57	1,16; 2.16; 0.16	1,16; 12.59; **≤0.01**	1,16; 1.01; 0.33	1,16; 12.96; **≤0.01**	1,16; 0.03; 0.86	1,16; 0.00; 0.95

Photosynthetic pathway, PP. O_2_ concentration, [O_2_]. Interaction effect, PP:[O_2_]. Table shows degrees of freedom, *F*-value, and *P*-value. Significant (*a<* 0.05) *P*-values are shown in bold.

#### CO_2_ assimilation during induction is enhanced under 2% O_2_ in some species but suppressed in others

In order to test whether the presence or absence of photorespiration affected the activation of CO_2_ assimilation, both light treatments were also conducted under 2% O_2_ (**Figures 4D-F, J-L**). In *Flaveria*, the decrease in O_2_ concentration significantly increased the AUC of both C_3_ *F. cronquistii* and C_4_ *F. bidentis* during the first five minutes of induction (**Table 3** and **Figure 5A**), under both light intensities (*P* ≤ 0.01; C_3_ 26.1 ± 5.4 vs C_4_ 19.9 ± 2.6 µmol m^-2^ at PFD = 600 µmol m^-2^ s^-1^ and *P* ≤ 0.001; C_3_ 33.8 ± 6.3 vs C_4_ 23.1 ± 1.6 at PFD = 1500 µmol m^-2^ s^-1^). Interestingly, although the stimulating effect of 2% O_2_ on C_4_ *F. bidentis* was less pronounced for 5 – 10 min and 10 – 60 min, the effect was still significant across both periods under both light intensities, except for 5 – 10 min at PFD = 600 µmol m^-2^ s^-1^ (*P* = 0.08, **Table 3**). This suggests that photorespiration is insufficiently suppressed during induction in C_4_ *F. bidentis*, whereas in contrast, no change was observed for CO_2_ assimilation in steady state C_4_ *F. bidentis* under 2% O_2_ (**Figures 2A, D**). In *Cleome* no such enhancement of the photosynthetic response was observed in C_4_ *G. gynandra*. Instead, the significant interaction between photosynthetic pathway and O_2_ concentration from 0 – 5 min was primarily associated with a decrease in assimilated CO_2_ in C_3_ *T. hassleriana* and only a marginal increase in C_4_ *G. gynandra* AUC under 2% O_2_ compared to under 21% O_2_ (*P* ≤ 0.001). The negative effect of 2% O_2_ on AUC in C_3_ *T. hassleriana* was transiently observed from 0 – 10 min at PFD = 600 µmol m^-2^ s^-1^ and only from 0 – 5 min at PFD = 1500 µmol m^-2^ s^-1^. From 10 – 60 min AUC at 2% O_2_ in C_3_ *T. hassleriana* was similar to the AUC at 21% O_2_ under both light intensities. Thus, the stimulation of steady state CO_2_ assimilation by 2% O_2_ in this species was not observed under any of the transient conditions (**Table 3** and **Figure 5**). Suppression of carbon assimilation by low O_2_ was also observed during the start of induction in both C_3_ and C_4_ *Alloteropsis* species. The AUC from 0 – 5 min was reduced in C_3_ *A. semialata GMT* and C_4_ *A. semialata MDG* compared to AUC in 21% O_2_ under both light intensities, a significant effect (*P* ≤ 0.001) that persisted well into the 5 – 10 min period for PPFD = 600 µmol m^-2^ s^-1^ (*P* ≤ 0.02). However, by 10 – 60 min the effect of O_2_ was reversed in C_3_ *A. semialata GMT*, with AUC for this period being significantly higher than for 21% O_2_ (*P* = 0.03). For this period C_3_ *A. semialata GMT* also had a significantly higher AUC in 2% O_2_ than C_4_ *A. semialata MDG* (*P* ≤ 0.01; C_3_ 504.4 ± 57.5 vs C_4_ 116.4 ± 84.7 µmol m^-2^ at PFD = 600 µmol m^-2^ s^-1^, and *P* = 0.03; C_3_ 558.71 ± 94.6 vs C_4_ 285.2 ± 108.7 µmol m^-2^ at PFD = 1500 µmol m^-2^ s^-1^). Whereas the stimulating effect of 2% O_2_ on transient CO_2_ assimilation may be indicative of photorespiration as a negative factor during photosynthetic induction, the suppression of carbon assimilation found under 2% O_2_ for both *Alloteropsis* species as well as the *Cleome* C_3_ *T. hassleriana* could indicate photorespiration is not always detrimental to photosynthetic efficiency and may indeed support the activation of CO_2_ assimilation in some C_3_ and C_4_ species.

#### Transient decoupling between electron transport and carbon fixation during induction is more pronounced in C_4_ species and ameliorated by 2% O_2_

**Figure 6:**
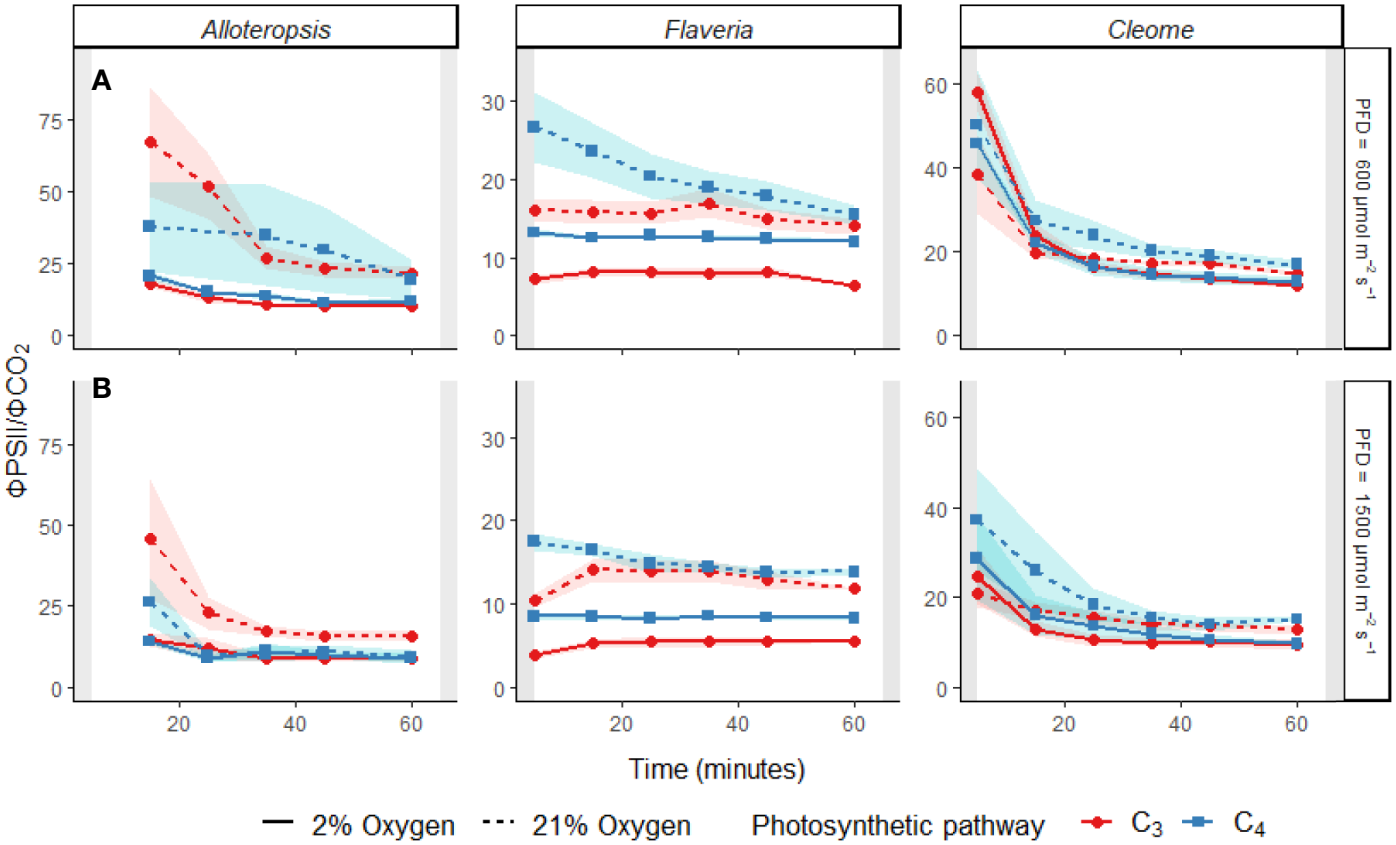
Line plots of the ΦPSII/ΦCO_2_ ratio during light induction at PFD = 600 µmol m^-2^ s^-1^ **(A)** and PFD = 1500 µmol m^-2^ s^-1^ **(B)** in phylogenetically linked C_3_ and C_4_ *Alloteropsis*, *Flaveria* and *Cleome* species. Plots show ΦPSII/ΦCO_2_ under 21% (dashed line) and 2% O_2_ (solid line). Values of ΦPSII/ΦCO_2_ were excluded if ΦCO_2_ values were negative, resulting in the 5 minute *Alloteropsis* values being excluded. Ribbons show standard error of the mean (n = 5).

During activation of CO_2_ assimilation, a temporary decoupling between the electron transport chain and photosynthetic carbon fixation in C_4_ species could occur due to the time needed to activate the C_3_ cycle, incomplete suppression of photorespiration due to an inactive CCM, or because of an increase in the energetic cost of carbon fixation *via* BS CO_2_ leakage. To look for evidence of transient decoupling during induction, ΦPSII/ΦCO_2_ ratios across the light induction period under each light and O_2_ condition were further analysed within each genus (**Figure 6** and **Table 4**).

**Table 4:** ANOVA table of the ΦPSII/ΦCO_2_ ratio during light induction for phylogenetically linked C_3_ and C_4_ *Alloteropsis*, *Flaveria* and *Cleome* species.

Light intensity (µmol m^-2^ s^-1^)	Genus		PP	t	[O_2_]	PP:[O_2_]	PP:t	O_2_:t	PP:[O_2_]:t
600	*Alloteropsis*	df; *F*-value; *P*-value	1,63; 0.11; 0.74	1,63; 28.69; **≤0.001**	1,63; 13.07; **≤0.001**	1,63;0.71;0.40	1,63; 0.62; 0.43	1,63; 5.98; **0.02**	1,63; 0.93; 0.34
	*Flaveria*	df;*F*-value;*P*-value	1,100; 52.64; **≤0.001**	1,100; 146.83; **≤0.001**	1,100; 6.96; **≤0.01**	1,100;0.13;0.73	1,100; 4.23; **0.04**	1,100; 6.60; **0.01**	1,100; 4.43; **0.04**
	*Cleome*	df;*F*-value;*P*-value	1,98; 0.69; 0.41	1,98; 7.71; **≤0.01**	1,98; 53.53; **≤0.001**	1,98;1.57;0.21	1,98; 0.00; 0.95	1,98; 0.04; 0.83	1,98; 0.30; 0.58
1500	*Alloteropsis*	df;*F*-value;*P*-value	1,70; 3.30; 0.07	1,70; 11.82; **≤0.001**	1,70; 12.50; **≤0.001**	1,70;3.90;**0.05**	1,70; 0.60; 0.44	1,70; 4.20; **0.04**	1,70; 0.46; 0.49
	*Flaveria*	df;*F*-value;*P*-value	1,101; 67.884; **≤0.001**	1,101; 478.57; **≤0.001**	1,101; 0.60;0.44	1,101;2.06;0.15	1,101; 9.33; **≤0.01**	1,101; 4.51; **0.03**	1,101; 2.91; 0.09
	*Cleome*	df;*F*-value;*P*-value	1,101; 4.14; **0.04**	1,101; 6.44; **0.01**	1,101; 27.65; **≤0.001**	1,101; 0.52;0.47	1,101; 3.06; 0.08	1,101; 00.00; 0.96	1,101; 0.64; 0.42

Photosynthetic pathway, PP. Time, t. O_2_ concentration, [O_2_]. Interaction effects, PP:[O_2_], PP:t, and PP:[O_2_]:t. Table shows degrees of freedom, *F*-value, and *P*-value. Significant (*a<* 0.05) *P*-values are shown in bold.

The effect of time was significant for all genera. All the C_4_ species showed higher ΦPSII/ΦCO_2_ at the start of induction under 21% O_2_, with values gradually decreasing as the leaves became more acclimated to the light conditions. The average ΦPSII/ΦCO_2_ ratio during induction was also noticeably higher than the steady state ΦPSII/ΦCO_2_ for all species, which ranged between 8-12 e^-^/CO_2_ depending on the species, indicating a significant transient decoupling during induction compared to steady state. In *Flaveria*, C_4_ *F. bidentis* had higher ΦPSII/ΦCO_2_ ratio than C_3_ *F. cronquistii* under all light and oxygen conditions, the complete opposite of steady state. This suggests the C_4_ pathway in *Flaveria* does have some features that make the activation of photosynthesis more energetically demanding at the onset of light induction. Notably, the interaction of O_2_ concentration and time also had a significant effect on ΦPSII/ΦCO_2_ (*P* = 0.01 at PFD = 600 µmol m^-2^ s^-1^, *P* = 0.01 at PFD = 1500 µmol m^-2^ s^-1^), since in 2% O_2_ the ratio decreased at an earlier point of induction than in 21% O_2_. During induction at PFD = 600 µmol m^-2^ s^-1^, the three-way interaction was significant (*P* = 0.04), reflecting the strong decrease in ΦPSII/ΦCO_2_ over time observed at 21% O_2_ in C_4_ *F. bidentis*. This decrease may reflect the progressive suppression of photorespiration by activation of the C_4_ CCM, since the same trend in ΦPSII/ΦCO_2_ was not present in C_3_ *F. cronquistii*, nor under 2% O_2_ when photorespiration would have been negligible.

In *Cleome*, C_3_ *T. hassleriana* also had a lower ΦPSII/ΦCO_2_ ratio than C_4_ *G. gynandra*, in another reversal of differences observed during steady state conditions. ΦPSII/ΦCO_2_ was significantly affected by time (*P* ≤ 0.01 at PFD = 600 µmol m^-2^ s^-1^, *P* = 0.01 at PFD = 1500 µmol m^-2^ s^-1^) as well as O_2_ concentration (*P* ≤ 0.001 at both light intensities), but in contrast to *Flaveria* no significant interaction was found between O_2_ and time (*P* = 0.21 at PFD = 600 µmol m^-2^ s^-1^, *P* = 0.47 at PFD = 1500 µmol m^-2^ s^-1^). Instead, in *Cleome* ΦPSII/ΦCO_2_ was marginally lower at 2% O_2_ than at 21% O_2_ across the induction period. The temporal decrease in ΦPSII/ΦCO_2_ was similar under both O_2_ concentrations, suggesting that the transient decoupling between electron transport and CO_2_ fixation was relatively insensitive to O_2_ in both *Cleome* species.

Finally, ΦPSII/ΦCO_2_ ratios in *Alloteropsis* were not significantly affected by a main effect of photosynthetic pathway (*P* = 0.74 at PFD = 600 µmol m^-2^ s^-1^, *P* = 0.07 at PFD = 1500 µmol m^-2^ s^-1^), again in contrast to steady state where C_4_ *A. semialata MDG* had lower ratios than C_3_ *A. semialata GMT*. However, the interaction between photosynthetic pathway and O_2_ was significant (*P* ≤ 0.001 at both light intensities), due to the fact that the O_2_ effect on ΦPSII/ΦCO_2_ was much less pronounced in C_4_ *A. semialata MDG* than in C_3_ *A. semialata GMT*. Similar to the *Flaveria* and *Cleome* results, a significant interaction between O_2_ concentration and time (*P* ≤ 0.001 at both light intensities) was also observed in *Alloteropsis*, with 2% O_2_ more significantly reducing ΦPSII/ΦCO_2_ during the start of induction than towards the end.

## Discussion

The presented experiments investigated the efficiency of photosynthesis during light induction in phylogenetically linked *Alloteropsis, Flaveria*, and *Cleome* C_3_ and C_4_ species. Steady state and photosynthetic induction responses to light were measured to evaluate relative differences between paired species – controlling for evolutionary distance allowed for better differentiation between the effects of photosynthetic pathway and species-specific variation. At the start of light induction C_4_ species had greater lag in CO_2_ assimilation than C_3_ species in all three comparisons (**Figure 5A**), confirming that the activation of CO_2_ assimilation is generally slower in C_4_ photosynthesis within the studied genera. However, the underlying reasons for this difference appeared to be genus specific. In C_4_ *Flaveria*, slower induction appeared to be explained at least in part by less efficient suppression of photorespiration, since 2% O_2_ resulted in increased CO_2_ assimilation and fewer transferred electrons per fixed CO_2_ (**Figure 6**). Although decreased photorespiratory electron sinks were also observable in C_4_ *Alloteropsis* and *Cleome* induction under 2% O_2_, there were no concurrent increases in CO_2_ assimilation (**Figure 5A**), implying alternative limiting factors were at play, such as C_3_ cycle activation. In C_3_ *Cleome* and both *Alloteropsis* species, 2% O_2_ actually suppressed activation of CO_2_ assimilation, suggesting that photorespiration may support the induction of photosynthesis in these species.

### Slower activation of CO_2_ assimilation during light induction in C_4_ versus C_3_ photosynthesis

In line with previously observed photosynthetic induction responses (Pearcy and Seemann, 1990; Pearcy, 1990; Sassenrath-Cole and Pearcy, 1992; Mott and Woodrow, 2000) a transient reduction in CO_2_ assimilation relative to steady state was observed in all species during light induction, with a more pronounced effect found in C_4_ species (**Figure 5**). Greater losses of photosynthetic efficiency have previously been observed in C_4_ grown under dynamic light in comparison to C_3_ species and linked to mechanisms involving photosynthetic induction (Kubásek et al., 2013). Further fluctuating light work on plants grown under constant light (including C_4_ *F. bidentis*) has also shown an increased lag in CO_2_ assimilation in C_4_ compared to C_3_ species following step-increases in light intensity (Li et al., 2021). Similarly, in a study comparing a selection of C_3_ and C_4_ grasses (Lee et al., 2022), a biphasic increase in assimilation during the low to high light transition was the most significant limitation in maize and big bluestem, again emphasizing the C_4_ lag in CO_2_ assimilation. However, both Li et al. (2021) and Lee et al. (2022) studies examined the efficiency of fully induced photosynthesis subsequently exposed to stepwise decreases and increases in light intensity, whereas the C_4_ lag-time when activation starts from darkness or from prolonged periods of low light may be even more pronounced.

During darkness or low light periods, stomatal closure could subsequently restrict photosynthetic assimilation during light induction due to a lack of coordination between CO_2_ influx and assimilation. However, g_sw_ appears to increase in tandem with decreases in C_i_, so stomatal opening does not seem to be the major source of limitation (**Figures 4C, F, I, L**). The lower g_sw_ found in C_4_ species during light induction is consistent with studies that show C_4_ species to have lower stomatal conductance and greater water use efficiency (Way et al., 2014; McAusland et al., 2016). It is worth noting that C_4_ monocots under dynamic light have been reported to have faster stomatal opening and closing than C_3_ monocots and dicots (Ozeki et al., 2022) yet stomatal kinetics in C_4_ *A. semialata MDG* instead appear to be slower, again emphasizing the importance of accounting for species or genus-specific phenomena. However, although CO_2_ availability can affect CO_2_ assimilation during induction, other biochemical limitations appeared to dominate the responses observed here, as discussed in more detail below. The different C_4_ decarboxylase pathways found across the three C_4_ species studied have distinct energetic demands per cell type. The NADP-ME subtype found in C_4_ *F. bidentis* and NAD-ME subtype in C_4_ *G. gynandra* require substantial transfer of reductant between M and BS cells (Ishikawa et al., 2016) and steeper metabolite gradients for CCM operation, whilst mixed NADP-ME-PEPCK CCM found in C_4_ *A. semialata MDG* can meet ATP and NADPH requirements more cell autonomously (Yin and Struik, 2021). Modelling simulations indicate that reduced metabolite concentrations may be required to sustain this C_4_ pathway (Wang et al., 2014a). The greater cell autonomy regarding energetic supply and demand found in C_4_ *A. semialata MDG* could suggest a capacity for faster activation of photosynthetic assimilation, yet in the light induction experiments CO_2_ assimilation in C_4_ *A. semialata MDG* lagged behind the other C_4_ species during the first ten minutes after starting light exposure (**Figure 5A, B**). In the following paragraphs we explore the different mechanisms underlying the slow activation of C_4_ photosynthesis across the three genera.

### Photorespiration during C_4_ photosynthetic induction, disadvantageous or beneficial?

Reduced CO_2_ assimilation during induction in C_4_ species has been hypothesised to derive from the need to build up C_4_ cycle intermediates leading to a lag in the efficient suppression of photorespiration (Sage and Mckown, 2006). If so, induction in C_4_ species when the photorespiratory pathway is suppressed by low O_2_ should result in an increase in photosynthetic carbon assimilation. This appeared to be confirmed in C_4_ *F. bidentis*, where CO_2_ assimilation during induction was higher under 2% O_2_ than under 21% O_2_ (**Figure 5**), in contrast to CO_2_ assimilation in C_4_ *F. bidentis* under steady state which showed no difference in A_CO2_ between O_2_ concentrations (**Figures 2A, D** and **Table 1**). Comparatively, the lack of an equivalent improvement in CO_2_ assimilation under 2% O_2_ in C_4_ *A. semialata GMT* and C_4_ *G. gynandra* suggests that in these species the activation of the C_3_ cycle (Sassenrath-Cole and Pearcy, 1992; Mott and Woodrow, 2000) could instead be the limiting factor.

Surprisingly, despite the stimulating effect of 2% O_2_ on steady state CO_2_ assimilation in C_3_ *A. semialata GMT* and C_3_ *T. hassleriana*, and lack of O_2_ sensitivity in C_4_ *A. semialata GMT* (**Figures 2A, D** and **Table 1**), CO_2_ assimilation in all three species during the first 10 min of light induction was lower in 2% O_2_ than in 21% O_2_ (**Figures 5A, B**). Reverse sensitivity to O_2_ in C_3_ species has been linked to limitation by the rate of triose phosphate utilisation (TPU) (Sharkey, 1985). Low O_2_ suppresses the net export of photorespiratory intermediates serine or glycine and limits endogenous pools of inorganic phosphate (P_i_), as the amino acids come from phosphorylated plastidic metabolites that when used up in the cytosol liberate P_i_ otherwise used in the glycerate-PGA conversion (McClain and Sharkey, 2019). Additionally, reduced rates of starch biosynthesis from triose phosphates, and phosphoglucose isomerase inhibition have been found in low O_2_ conditions (Dietz, 1985). The lower CO_2_ assimilation observed in 2% O_2_ compared to 21% O_2_ during induction in C_3_ *A. semialata GMT* and C_3_ *T. hassleriana* could thus be due to 2% O_2_ causing suboptimal stromal phosphate levels, thereby transiently exacerbating TPU limitation. It remains unclear whether C_4_ species suffer from TPU limitation (Zhou et al., 2019), or whether alternative mechanisms may be involved.

The photorespiratory pathway has previously been suggested to help prime the C_4_ cycle by providing a carbon source from which to build C_3_ and C_4_ metabolite pools (Kromdijk et al., 2014; Stitt and Zhu, 2014; Schlüter and Weber, 2020; Fu and Walker, 2022; Medeiros et al., 2022). In C_4_ species, photorespiration could help establish CCM metabolic gradients through interconversion of 3-phosphoglyceric acid (3-PGA) and PEP (Arrivault et al., 2016), and in plants with NADP-ME decarboxylase, such as mixed subtype NADP-ME PEPCK C_4_ *A. semialata GMT*, models suggest photorespiration could support the activation of redox-regulated C_3_ enzymes and contribute to the formation of C_3_ cycle intermediates in the BS through the triose phosphate transporter (TPT) (Weber and Von Caemmerer, 2010; Wang et al., 2014b). In C_3_ photosynthesis, beyond its photoprotective role (Kozaki and Takeba, 1996), photorespiration has been shown to enhance CO_2_ fixation through the assimilation of nitrogen (Busch et al., 2018). A recent metabolomic analysis in maize suggested that photorespiratory intermediates may also provide this supporting role in C_4_ species (Medeiros et al., 2022).

### Decoupling between electron transport and photosynthesis: Alternative electron sinks and BS leakiness

Particularly at the start of light induction, C_3_ and C_4_ species had significantly higher ΦPSII/ΦCO_2_ ratios (**Figure 6**) than during steady state measurements (**Table 1**), indicating less of the reducing power of the electron transport chain was going towards photosynthetic carbon fixation. This was particularly prominent in the C_4_ species, both in absolute values and relative to steady state, where C_4_ plants had either lower (C_4_ *Alloteropsis* and *Flaveria*) or equal (C_4_ *Cleome*) ΦPSII/ΦCO_2_ values compared to their C_3_ counterparts. The build-up of metabolite pools to establish sufficient concentration gradients between M and BS cells required for the efficient operation of C_4_ photosynthesis seems a likely contributing factor increasing ΦPSII/ΦCO_2_ ratios in C_4_ photosynthesis. However, the change in ratio could also be due to a variety of alternative electron sinks having greater presence during induction and drawing electrons away from the C_3_ cycle.

Not surprisingly, reductions in ΦPSII/ΦCO_2_ ratio under 2% O_2_ were observed in all C_3_ species as well as in C_4_ *F. bidentis*, showing the importance of photorespiration as an electron sink. Although a gradual decrease in ΦPSII/ΦCO_2_ across time was observed during induction in both *Alloteropsis* and *Cleome* C_4_ species (**Figure 5**), in contrast to C_4_ *F. bidentis* the temporal changes were not O_2_ sensitive. An alternative electron sink to photorespiration could be the Mehler reaction, which reduces O_2_ in the chloroplast to hydrogen peroxide and has been suggested to play a role in C_3_ and C_4_ photosynthesis (Sagun et al., 2021). Suppression of the Mehler reaction under 2% O_2_ could be consistent with the small reductions in CO_2_ assimilation observed in both *Alloteropsis* species and C3 *T. hassleriana*, as the Mehler reaction supports ATP formation and the activity of related enzymes has been found to increase when photosynthesis is impaired (Fryer et al., 1998). However, evidence to support a significant contribution of the Mehler reaction to high rates of photosynthesis in both C_3_ and C_4_ species is generally lacking (Driever and Baker, 2011).

A transient increase in BS leakiness could be an alternative contributing factor to the elevated energetic cost of CO_2_ assimilation during induction in C_4_ *A. semialata* and C_4_ *G. gynandra* that accounts for the lack of O_2_ sensitivity. A lag in activation of the C_3_ cycle following light exposure would result in an imbalance between the C_3_ and C_4_ cycles and greater leakage of CO_2_ from the BS due to the CCM over-pumping, reducing quantum efficiency by requiring more ATP per CO_2_ fixed (Kromdijk et al., 2014, Sage and McKown, 2006). Transient isotope discrimination measurements on sorghum and maize during the first 10 min following a step-increase in light intensity suggested that bundle sheath leakiness could be 60% higher than steady state (Kubásek et al., 2013; Wang et al., 2022) and remain elevated for up to 30 min. This seems consistent with the timing of the decrease in ΦPSII/ΦCO_2_ during induction in the *Alloteropsis* and *Cleome* C_4_ species. Thus, activation of CO_2_ assimilation in these species may be limited by activation of the C_3_ cycle, whereas the stable ΦPSII/ΦCO_2_ values in C_4_ *F. bidentis* under 2% O_2_ suggest C_3_ cycle activation is faster than the CCM in this species.

## Conclusion

This study confirms C_4_ photosynthesis experiences greater lag than C_3_ photosynthesis during light induction – the greater depression of CO_2_ assimilation in C_4_ species was independently found in three evolutionary divergent comparisons of phylogenetically linked C_3_ and C_4_ species, providing experimental support for previous hypotheses and observations of less efficient photosynthetic induction in C_4_ photosynthesis (Sage and Mckown, 2006; Kubásek et al., 2013; Slattery et al., 2018; Li et al., 2021; Sales et al., 2021). Despite the generally slower induction of CO_2_ assimilation found in all C_4_ species in comparison to their C_3_ pairs, the underlying mechanisms to explain these differences were distinctly different – less effective suppression of photorespiration seemed to underlie the reduction in CO_2_ assimilation in C_4_ *Flaveria* whereas delayed activation of the C_3_ cycle appeared to be the limiting factor in C_4_ species in *Alloteropsis* and *Cleome*, where a potential supporting role for photorespiration in photosynthetic induction was also identified. The substantial variation observed between and across phylogenetic pairs during both steady state and light induction measurements underscores the crucial importance of controlling for evolutionary distance when studying differences between photosynthetic pathways.

## Data availability statement

The raw data supporting the conclusions of this article will be made available by the authors, without undue reservation.

## Author contributions

JK conceived the study. JK, LAC and ACB designed the experiments. LAC carried out all experiments, data analysis and interpretation, and drafted the manuscript. RLV helped with the 2% O_2_ experimental setup and provided support with gas exchange experiments. ELB procured the initial plant material. CRGS helped with data interpretation. All authors contributed to the article and approved the submitted version.
