# Corrigendum: Activation of CO_2_ assimilation during photosynthetic induction is slower in C_4_ than in C_3_ photosynthesis in three phylogenetically controlled experiments

**DOI:** 10.3389/fpls.2023.1188404

**Published:** 2023-03-29

**Authors:** Lucía Arce Cubas, Richard L. Vath, Emmanuel L. Bernardo, Cristina Rodrigues Gabriel Sales, Angela C. Burnett, Johannes Kromdijk

**Affiliations:** ^1^ The University of Cambridge, Department of Plant Sciences, Cambridge, United Kingdom; ^2^ University of the Philippines Los Baños, Institute of Crop Science, College of Agriculture and Food Science, College, Laguna, Philippines

**Keywords:** C_4_ photosynthesis, C_3_ photosynthesis, photosynthetic induction, CO_2_ assimilation, photorespiration, non-steady state, light response

In the published article, there was an error in the affiliation(s) for Emmanuel L. Bernardo. As well as having affiliation 1, they are also affiliated to University of the Philippines Los Baños, Institute of Crop Science, College of Agriculture and Food Science, College, Laguna, Philippines.

The authors apologize for this error and state that this does not change the scientific conclusions of the article in any way. The original article has been updated.

