# Supplementary material for: Activation of CO2 assimilation during photosynthetic induction is slower in C4 than in C3 photosynthesis in three phylogenetically controlled experiments
Source: Front Plant Sci. 2023 Jan 4;13:1091115. doi: 10.3389/fpls.2022.1091115 (PMC9848656; doi:10.3389/fpls.2022.1091115)
Supplement: Supplementary file 1 [file Table_1.docx]

Supplementary Material

**Supplementary material 1:** Leaf absorptance values of phylogenetically linked C3 and C4 *Alloteropsis*, *Flaveria* and *Cleome* species from the blue (470 nm) and red (630 nm) wavelengths of the actinic light source used in experiments (6400-40 Leaf Chamber Fluorometer, LI-COR), measured with an integrating sphere. Means and standard error of the mean are shown (n = 5).

| **Genus** | **Species** | **Abs_470** | **Abs_630** |
| --- | --- | --- | --- |
| *Alloteropsis* | C3 *A. semialata GMT* | 0.90±0.02 | 0.88±0.02 |
|  | C4 *A. semialata MDG* | 0.87±0.02 | 0.81±0.02 |
| *Flaveria* | C3 *F. cronquistii* | 0.87±0.04 | 0.79±0.05 |
|  | C4 *F. bidentis* | 0.94±0.00 | 0.89±0.01 |
| *Cleome* | C3 *T. hassleriana* | 0.94±0.00 | 0.88±0.01 |
|  | C4 *G. gynandra* | 0.85±0.03 | 0.78±0.03 |
